# 6-year treatment follow-up with an extended-release alkaline formulation (Sibnayal^®^) in primary distal renal tubular acidosis

**DOI:** 10.1186/s13023-025-03953-4

**Published:** 2025-08-13

**Authors:** Aurélia Bertholet-Thomas, Aurélie De Mul, Julie Bernardor, Gwenaëlle Roussey-Kesler, Ludmila Podracka, Robert Novo, François Nobili, Bertrand Knebelmann, Jérôme Harambat, Emilija Golubovic, Olivia Boyer, Massimo Di Maio, Mathilde Cailliez, Véronique Baudouin, Laure Chidler, Véronique Leblanc, Justine Bacchetta

**Affiliations:** 1 Centre de Référence des Maladies Rénales Rares– MAREGE– Hôpital Femme Mère Enfant, Hospices Civils de Lyon– Filière ORKID (Orphan Kidney Diseases), ERK-Net (The European Rare Kidney Disease Network), Lyon, France; 2 Hôpital l’Archet, Service de Rhumatologie Pédiatrique et Médecine Interne de l’enfant, CHU de Nice, Nice, France; 3 Hôpital Mère-Enfant, Clinique Médicale Pédiatrique, CHU de Nantes, Unité de Néphrologie et Hémodialyse Pédiatrique, Nantes, France; 4 Department of Pediatrics, National Institute of Children’s, Bratislava, Slovakia; 5 Service de Néphrologie Pédiatrique, Hôpital Jeanne de Flandre, CHRU de Lille, Lille, France; 6 Hôpital Jean Minjoz, CHU de Besançon, Service de Pédiatrie 2, Besançon, France; 7 Service de Néphrologie adultes, Hôpital Necker, APHP, Paris, France; 8 Service de Pédiatrie, Hôpital Pellegrin- Enfants, CHU de Bordeaux, Bordeaux, France; 9 Klinički Centar Niš, Niš, Serbia; 10 APHP, Service de Néphrologie Pédiatrique, Hôpital Necker-Enfants Malades, Paris, France; 11 Service de Réanimation Néonatale et Néonatologie, CHU de Nîmes, Nîmes, France; 12 AP-HM, Service de Pédiatrie Multidisciplinaire, Hôpital de la Timone, Marseille, France; 13 Service de Néphrologie Pédiatrique, Hôpital Universitaire Robert Debré-APHP, Paris, France; 14 ADVICENNE, Paris, France; 15 Centre de référence des maladies rares du métabolisme du calcium et du phosphate-Hospices, Civils de Lyon-OSCAR-ERN BOND, Lyon, France

**Keywords:** Bone metabolism, Bone mineral density, Distal renal tubular acidosis, Estimated glomerular filtration rate, Growth, Alkalinizing therapy

## Abstract

**Background:**

Distal renal tubular acidosis (dRTA) is a rare disease characterized by hyperchloremic metabolic acidosis affecting growth, bone and kidney health.

**Methods:**

The aim of B22CS study was to evaluate long-term safety and efficacy (anthropometric/pubertal, tubular damages/kidney function, bone biomarkers, compliance assessments) of Sibnayal^®^, a prolonged-release alkalinizing formulation with twice daily dosing, in children and adults with dRTA. All patients were previously included in the pivotal B21CS study, so were already receiving Sibnayal^®^ when included in B22CS open-label follow-up study.

**Results:**

A total of 30 patients with primary dRTA (mean age:10.6 ± 6.0 years) entered this long-term study (average of 6 years). At inclusion, most patients had adequate metabolic control, normal kidney function and height. Sibnayal^®^ was well tolerated over the study duration.The most frequent adverse event was hypovitaminosis D (13 patients). Causality to treatment was reported for only 4% of all TEAEs (6 patients) and were mostly gastrointestinal. All adverse events resolved without treatment discontinuation. Sibnayal^®^ allowed a sustained control of metabolic acidosis as plasma bicarbonate level was 22.0 ± 3.2 mmol/L at baseline versus 22.6 ± 2.5 mmol/L at the End of Follow-up (EoF), p = NS. From baseline to EoF, mean Z-score height significantly increased (-0.6 ± 1.0 to -0.3 ± 1.0, *p* = 0.03), without significant change in weight and body mass index. Kidney function remained stable from baseline to EoF: estimated glomerular filtration rate = 105 ± 17 and 104 ± 20 mL/min/1.73m^2^, respectively, p = NS. Urinary ratios: Calcium/Creatinine (UCa/UCr), Citrate/Creatinine (UCi/UCr), Calcium/Citrate (UCa/UCi) were not significantly different between baseline and EoF (p = NS). Mean lumbar bone mineral density Z-score significantly increased from baseline (-1.1 ± 1.0) to EoF (-0.8 ± 1.0), *p* = 0.005, with significant improvement between baseline and EoF in pre- and post-pubertal patients (*p* = 0.035 and *p* < 0.001, respectively), whilst it was maintained in pubertal patients (p = NS).

**Conclusion:**

Long-term data support the good safety and efficacy profile of Sibnayal^®^ in the treatment of dRTA with adequate control of metabolic acidosis, stable kidney function and significant positive long-term clinical outcomes.

**Supplementary Information:**

The online version contains supplementary material available at 10.1186/s13023-025-03953-4.

## Key learning points

**What was known:**

The medical management of dRTA consists in the correction of metabolic acidosis to prevent long-term complications on growth, bone metabolism and kidney function. Growth and kidney function were shown to be correlated to metabolic control. Only half of patients had adequate metabolic control. More than 80% of adult patients had CKD KDIGO ≥ 2.

**This study adds:**

For the first time, a long-term follow-up study provides safety and efficacy data of Sibnayal^®^, an alkali medication with long-lasting release allowing 2 intakes per day. This study supports a long-term adequate control of metabolic acidosis with Sibnayal^®^, along with significant clinical outcomes on growth, kidney function and bone metabolism.

**Potential impact:**

The main challenge for physicians is to prevent long-term complications of dRTA by better controlling metabolic acidosis. Sibnayal^®^ as an innovative prolonged-release alkalinizing formulation with twice daily dosing, adapted for children, contributes to improving current medical management, clinical outcomes and quality of life of dRTA patients.

**Trial Registration Number:**

Registered as EudraCT 2013-003828-36 on September 03, 2013.

## Background

Distal renal tubular acidosis (dRTA) is a rare tubular disease that can be inherited (with sensorineural hearing impairment in some genetic forms) and may also be acquired especially in adults [1]. The disorder is characterized by hyperchloremic metabolic acidosis with normal plasma anion gap, usually associated with hypokalemia. It induces negative effects on bone health and growth, such as stunting and failure to thrive, rickets in children, and osteomalacia in adults, which can be corrected with adequate control of metabolic acidosis [2–5]. Hypercalciuria and hypocitraturia observed in dRTA lead to nephrocalcinosis, nephrolithiasis contributing to chronic kidney disease (CKD) [2–5]. Concerning inherited dRTA, recent European guidelines from the European Rare Kidney Disease Reference Network (ERKNet) and the European Society of Paediatric Nephrology (ESPN) have delineated the management of such patients [1].

Alkalinizing therapy aims at normalizing bicarbonate levels to control metabolic acidosis and at preventing long-term complications [5]. The current Standard of Care (SoC) consists of alkalinizing products, administered 3 to 6 times per day. The low gastrointestinal tolerability and bad taste of most alkali treatments, leading to poor adherence, explain that only half of patients have an adequate control of metabolic acidosis [5].

As an alternative, Sibnayal^®^, a prolonged-release granules and tasteless formulation of potassium citrate and potassium bicarbonate, allows a 12 h-effect after a single dose, and is well tolerated [6]. In adult and pediatric patients with dRTA, Sibnayal^®^ has demonstrated an improved control of plasma bicarbonate levels, a surrogate marker of metabolic acidosis, over SoC treatments currently available, together with good acceptability and tolerability [7]. After 24 months of treatment with Sibnayal^®^, safety, tolerability, efficacy, and compliance to treatment in these patients were maintained [8].

The aims of the B22CS study were to evaluate the long-term safety and efficacy of Sibnayal^®^, in pediatric and adults dRTA patients, with an average follow-up of 6 years.

## Materials and methods

### Study design

**Fig. 1:**
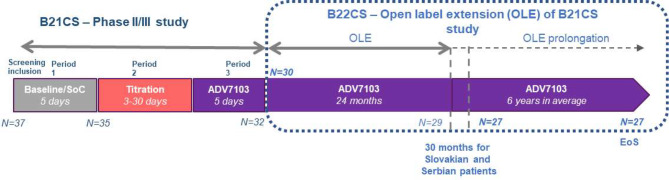
Clinical design of Arena 1 program including B21CS and B22CS studies. The B21CS, Phase II/III study enrolled 37 patients and included three treatment periods: baseline/SoC, Sibnayal^®^ titration period, and fixed-dose Sibnayal^®^ period. A total of 30 patients from B21CS study entered the OLE B22CS study and 27 patients completed the study with an average of 6 years of treatment with Sibnayal^®^ (30 months for Slovakian and Serbian patients). *EoS*, End of Study; *N*, number of evaluated patients; *OLE*, open label extension; *SoC*, Standard of Care

As previously reported, all study patients had primary dRTA [8]. Patients were divided into 4 age-groups: adults ≥ 18 years old, teenagers [12–18 years old], children [4–12 years old], and infants/toddlers [6 months-4 years old]. They were previously enrolled in a multicenter phase II/III trial, B21CS (EudraCT 2013-002988-25) having demonstrated the non-inferiority and then the superiority of Sibnayal^®^ as compared to SoC to maintain plasma bicarbonate level in the normal range [7]. These patients were then invited to participate in the multicenter, single-arm, open-label, follow-up B22CS study (EudraCT 2013-003828-36). At baseline visit (last visit of phase II/III trial), all patients were already treated by Sibnayal^®^. The study was first analyzed after a short follow-up period of 24 months [8] and we present here the results of the long-term 6-year follow-up period with yearly visits (Fig. 1). All patients were included in the efficacy and safety analyses. Patients received doses of Sibnayal^®^ twice daily, adjusted to reach normal plasma bicarbonate levels, as defined in each local laboratory and assessed by each clinical Investigator. Blood extractions for biochemical determinations were performed before or after the morning treatment intake (analyses have been duplicated on all values and on the subset of values issues from blood test done before the morning intake for some parameters, such as bicarbonatemia.

### Safety evaluation

Treatment emergent adverse events (adverse events occurring under the course of the study, TEAEs) were recorded with their severity and relationship to the treatment. The number and percentage of patients with TEAEs by system organ class and preferred term, according to the Medical Dictionary for Regulatory Activities (MedDRA, Version 18.0), were calculated.

### Anthropometric and pubertal evaluations

Height (cm), weight (kg), and Body mass index (BMI) (kg/m^2^) were measured at yearly visits and expressed as Z-scores for age according to World Health Organization standards [9, 10]. Height, weight and BMI Z-scores <-2.0 were considered for failure to thrive and stunting [11, 12].

Genetic target height was determined from parents’ final height, according to the American College of Medical Genetics practice guideline [13]. The Estimated Adult Stature (EAS) was calculated at baseline, Month 24, Month 48 and EoS as the height at Tanner stage 5 or the extrapolated height at 19 years (considering the Z-score at the time of evaluation). The EAS was considered normal when it was within the range target height ± 10 cm for boys and target height ± 9 cm for girls [14].

Pubertal maturity was evaluated according to Tanner stage standard tables containing the age percentiles for each stage in the reference population [15–17].

### Evaluation of kidney function

The following parameters were measured in local laboratories: plasma bicarbonate, kalemia and creatininemia, urinary pH (evaluated by glass electrode pH-meter), ratio of tubular maximum reabsorption of phosphate to glomerular filtration rate (TmP/GFR), urine calcium/creatinine ratio (UCa/UCr), urine citrate/creatinine ratio (UCi/UCr), and urine calcium/citrate ratio (UCa/UCi). Normal ranges were provided from published data depending on patient age and gender [18–21].

The estimated glomerular filtration rate (eGFR) was calculated according to the CKiD Under 25 (CKiDU-25) Eq. [22]. Decreased kidney function was considered when eGFR was < 90 mL/min/1.73 m^2^ with CKD stages according to Kidney Disease Improving Global Outcomes guidelines [23].

The risk of lithogenesis was evaluated with a threshold of UCa/UCi above 3.0 mmol/mmol and UCa/UCr above normal range according to age [24].

Crystalluria analyses were performed in local laboratories with focus on amorphous carbonated calcium phosphate (ACCP) crystals that are frequent in case of alkaline urine whatever its cause including dRTA. Crystalluria was evaluated by the examination of a well-homogenized fresh morning urine sample set in a Malassez cell by light microscopy.

The presence or absence of nephrocalcinosis (without grading) and nephrolithiasis was assessed at each visit mainly by kidney ultrasounds and possibly computed tomography (CT)-scan when it was available for adult patients.

### Bone biomarkers assessment

Rickets and osteomalacia were determined following investigator’s clinical judgement, based on clinical signs (diffuse bone and joint pain, myalgia, muscle weakness, abnormal posture, abnormal stature, walking difficulty), radiologic signs (abnormal bone aspect, bone deformation, pseudofracture) and biochemical signs (abnormal values of plasma bone alkaline phosphatases (bALPs), calcium and phosphate). Bone and phosphate/calcium metabolism biomarkers were assessed up to Month 48 in a central laboratory for 1-25-dihydroxy-vitamin D (1-25-OH-vit-D, normal range: 69–200 pmol/L), bALPs, (normal values depending on age and gender), parathyroid hormone (PTH, normal range: 5.5–38.4 ng/L). In contrast, 25-hydroxy-vitamin D (25-OH-vit-D), plasma phosphate and calcium were performed in local laboratories. Plasma phosphate and bALPs were expressed as Z-scores for age and gender [25, 26].

Bone Mineral Density (BMD, g/cm^2^) was measured yearly in three skeletal regions (lumbar spine, hip and whole body) using dual-energy X-ray absorptiometry (DXA) and expressed as Z-score. Only lumbar spine area results are presented as this is the only relevant skeletal area for both pediatric and adult populations. Values of Z-score <-2.0 SD were considered below the normal range according to the recommendations of the International Society for Clinical Densitometry [27].

### Evaluation of compliance to treatment

Patients were asked to return their unused treatment units (sachets) at each visit to calculate the proportion of treatment that had been taken versus what should have been taken according to study protocol. When patients did not return unused sachets, compliance was estimated by questioning the patient and/or his/her family while considering laboratory results. In case of doubt, treatment was considered as not taken by the patient. Treatment compliance was qualified “poor” when compliance rate was < 50%, “average” at 50 to < 75%, “good” at 75 to 90%, and “excellent” at > 90%.

### Statistical analyses

The final data analysis included data collected from November 2014 to the end of the study dated October 2021. All patients receiving at least one dose of Sibnayal^®^ and with at least one efficacy assessment were included in the efficacy analysis set.

Safety and efficacy data and their change from baseline (when appropriate) were summarized over time using descriptive statistics by age group (according to age at baseline) or considering pooled pediatric patients and the overall patient population.

The originally planned statistical analyses B22CS studies were mainly descriptive from baseline to End of Study (EoS, last visit in the study), except for a repeated measures model on change from baseline adjusted on baseline for lumbar spine BMD Z-scores. All other statistical tests and associated figures were performed as post-hoc analyses.

For these post-hoc analyses, an End of Follow-up (EoF) value corresponding to the last available patient value for each parameter was computed. The EoF values were compared to baseline values from the B22CS study, to assess the evolution of parameters between first and last measurements. The value measured during the EoS visit was used when assessing the evolution of a parameter over time throughout the study.

Data were described as proportions or mean (± standard deviation, ±SD) according to the nature of the parameter. To compare two quantitative values (mean at baseline versus EoF for instance), a paired Student’s t-test or a Wilcoxon signed-rank test was used depending on if the data is normally distributed or not, respectively (normality was assessed using Skewness and Kurtosis values as well as graphical checks). When proportions were compared (number of patients in normal range at baseline versus EoF for instance), a McNemar’s test (when two categories are involved) or a symmetry test (when more than two categories are involved) was used. To evaluate the evolution of a parameter over time, a repeated measures model on change from baseline, adjusted on baseline, was performed. Values of mean least squares and 95% confidence interval (CI) were reported for the difference between each visit and baseline. All statistical tests were two-sided and carried out at the 5% level of significance. These tests were performed for exploratory purposes only and no adjustment for multiplicity was performed. Most tests are displayed with a graphical representation.

Analyses were performed using SAS^®^ software, Version 9.4 (SAS Institute Inc., Cary, NC, USA).

## Results

Main data of B22CS patients are summarized in Table 1.

**Table 1:** Main anthropometric and biological data of baseline, eof, month 48 and EoS of B22CS

	B22CS baseline	B22CS EoF	*p*-value: comparison Baseline vs. EoF
**Age** (years) Mean ± SD SEM Median Min-max	*N* = 30 10.6 ± 6.0 1.1 9.5 2.0–21.0	*N* = 30 16.4 ± 6.0 1.1 15.5 7.8–28.0	NA
**Height Z-score** Mean ± SD SEM Median Min-max	*N* = 28 -0.6 ± 1.0 0.2 -0.7 -3.3-1.5	*N* = 28 -0.3 ± 1.0 0.2 -0.3 -2.2-1.5	*p* = 0.032
**Weight Z-score** Mean ± SD SEM Median Min-max	*N* = 29 0.2 ± 1.5 0.3 0.0 -2.5-3.2	*N* = 29 0.6 ± 1.3 0.2 0.4 -1.7-3.3	*p = NS*
**BMI Z-score** Mean ± SD SEM Median Min-max	*N* = 28 0.3 ± 1.5 0.3 0.3 -2.3-3.3	*N* = 28 0.2 ± 1.4 0.2 0.3 -2.3;2.7	*p = NS*
**EAS** **N patients in normal range (%)**	*N* = 26 20 (77%)	*N* = 26 21 (81%)	*p = NS*
**Bicarbonatemia** (mmol/l) Mean ± SD SEM Median Min-max	*N* = 30 22.0 ± 3.2 0.6 22.0 13.7–29.0	*N* = 30 22.6 ± 2.5 0.5 22.9 17.0–27.0	*p = NS*
**Kalemia** (mmol/l) Mean ± SD SEM Median Min-max	*N* = 22 3.8 ± 0.5 0.1 3.8 2.7–4.7	*N* = 22 3.7 ± 0.4 0.1 3.7 2.9–4.2	*p = NS*
**eGFR** (mL/min/1.73 m^2^) Mean ± SD SEM Median Min-max **KDIGO stage**,** N patients (%)** Stage 1 Stage 2 Stage 3 Stage 4	*N* = 23 105 ± 17 3.5 100.5 79.0-149.8 20 (87%) 3 (13%) 0 (0%) 0 (0%)	*N* = 23 104 ± 20 4.1 104.5 76.5-158.1 17 (74%) 6 (26%) 0 (0%) 0 (0%)	*p = NS* *p = NS*
**UCa/UCr** **N patients in normal range**,** (%)**	*N* = 27 27 (100%)	*N* = 27 24 (89%)	*p = NS*
**UCi/UCr** **N patients in normal range**,** (%)**	*N* = 20 7 (35%)	*N* = 20 9 (45%)	*p = NS*
**UCa/UCi** **N patients in normal range** (UCa/UCi < 3 mmol/mmol), (%)	*N* = 20 9 (45%)	*N* = 20 6 (30%)	*p = NS*
**TmP/GFR** (mmol/l) Mean ± SD SEM Median Min-max	*N* = 20 1.5 ± 0.2 0.0 1.5 1.1–1.8	*N* = 20 1.3 ± 0.3 0.1 1.3 0.6–1.7	*p* = 0.001
**UpH** N patients (%) < 7.0 7.0–8.0 > 8.0	*N* = 23 2 (9%) 16 (70%) 5 (22%)	*N* = 23 0 (0%) 16 (70%) 7 (30%)	*p = NS*
**Crystalluria** N patients (%) **ACCP crystal** N patients (%)	*N* = 17 9 (53%) 8 (47%)	*N* = 17 6 (35%) 6 (35%)	*p = NS* *p = NS*
**Calcemia** (mmol/l) Mean ± SD SEM Median Min-max	*N* = 26 2.38 ± 0.10 0.0 2.4 2.2–2.5	*N* = 26 2.42 ± 0.08 0.0 2.4 2.2–2.6	*p* = 0.048
**Lumbar spine BMD (**Z-score) Mean ± SD SEM Median Min-max **N patients in normal range (>-2SD) (%)**	*N* = 24 -1.1 ± 1.0 0.2 -1.1 -3.2-0.6 17 (71%)	*N* = 24 -0.8 ± 1.0 0.2 -0.8 -2.7-1.1 21 (87%)	*p* = 0.005 *p = NS*
	**B22CS baseline**	**B22CS** **Month 48**	**p-value: Comparison Baseline vs. Month 48**
**Phosphatemia** (Z-score) Mean ± SD SEM Median Min-max	*N* = 23 -0.7 ± 0.7 0.1 -0.7 -2.0-1.0	*N* = 23 -1.4 ± 1.1 0.2 -1.2 -3.5-0.4	*p* = 0.005
**bALPs** (Z-score) Mean ± SD SEM Median Min-max	*N* = 9 0.9 ± 2.2 0.7 -0.0 -1.3-4.9	*N* = 9 0.7 ± 1.3 0.4 0.3 -0.6-3.0	*p = NS*
**25-OH-vit-D** (nmol/L) Mean ± SD SEM Median Min-max	*N* = 9 55.0 ± 33.7 11.2 41.0 25.0-127.3	*N* = 9 51.0 ± 19.2 6.4 52.0 33.0–93.0	*p = NS*
**1-25-OH-vit-D** (pmol/L) Mean ± SD SEM Median Min-max	*N* = 9 164.6 ± 54.1 18.0 172.0 100.0-230.0	*N* = 9 158.6 ± 36.3 12.1 159.0 111.0-200.0	*p = NS*
**PTH** (ng/L) Mean ± SD SEM Median Min-max **N patients in normal range (%)**	*N* = 15 15.6 ± 8.2 2.13 11.7 8.2–37.2 15 (100%)	*N* = 18 16.6 ± 6.6 1.55 16.6 7.2–37.8 18 (100%)	NA
	**B22CS baseline**	**EoS**	
**Nephrocalcinosis** N patients (%)	*N* = 29 25 (86%)	*N* = 25 23 (92%)	NA
**Nephrolithiasis** N patients (%)	*N* = 29 6 (21%)	*N* = 25 11 (44%)	NA
**Sibnayal**^®^**dose** (CK-BK PR, mEq/kg/d) Mean ± SD SEM Median Min-max	*N* = 29 3.4 ± 1.7 0.3 3.2 1.1-8.0	*N* = 26 2.3 ± 1.1 0.2 2.1 1.0-5.1	

*1-25OHvitD*, 1-25dihydroxyvitamin D; *25OHvitD*, 25hydroxyvitamin D; *ACCP*, amorphous carbonated calcium phosphate; *bALP*, blood alkaline phosphatase; *BMD*: bone mineral density; *BMI*, body mass index; *EAS*, estimated adult stature; *eGFR*, estimated glomerular filtration rate; *EoF*, End of Followup; *EoS*, End of Study; KDIGO, Kidney Disease: Improving Global Outcomes; *Max*, maximum; *Min*, minimum; *N*, number of patients; *NS*, nonsignificant; *PTH*, parathyroid hormone; *SD*, standard deviation; *SEM*, standard error of the mean; *SoC*, standard of care; *TMP/GFR*, ratio of renal tubular reabsorption of phosphorus to glomerular filtration rate; *UCa/UCi*, urinary ratio of calcium/citrate; *UCa/UCr*, urinary ratio of calcium/creatinine; *UCi/UCr*, urinary ratio of citrate/creatinine; UpH, urinary pH, CK-BK PR, potassium citrate and potassium bicarbonate prolonged release

### Patients’ characteristics

A total of 30 patients (6 adults, 8 teenagers, 13 children and 3 infants), all presenting with primay dRTA, entered the long-term study, 27 of whom had data collected beyond Month 30 (Fig. 1). The mean age (years) of patients were 10.6 ± 6.0 (min-max: 2–21) at baseline and 16.4 ± 6.0 (min-max: 7.8–28.0) at EoF, respectively. Mean and median durations (years) of follow-up were 5.8 ± 1.3 and 6.3 (min-max:1.1–6.8), respectively (Table 1). A total of 22 patients (73%) underwent genetic analysis, that found pathogenic variants in 21 patients (ATP6V0A4, *N* = 9, ATP6V1B1 *N* = 12) while the genetic mutation was not identified for 1 patient. Hearing impairment was reported in 20 patients, including all 6 adults.

### Safety

Over the course of the study, a total of 309 TEAEs were experienced by 29 patients. The most frequently reported TEAEs were vitamin D deficiency (i.e. hypovitaminosis D) (13 patients, 43.3%), hypokalemia (8 patients, 26.7%), iron deficiency (7 patients, 23.3%) and decreased appetite (2 patients, 6.7%). Of all TEAEs, 13 (4%) TEAEs were related to treatment, reported in 6 patients (20%). The majority of these related TEAEs were gastrointestinal disorders (*N* = 11), reported in 5 patients (16.7%), and were of mild or moderate intensity. All adverse events resolved without discontinuation of the treatment. No serious adverse events related to the treatment were reported (Supplemental Table S1).

### Changes in pubertal maturity and growth

Pubertal maturity was normal in most pediatric patients throughout the study (> 90%). Overall, 11/23 pediatric patients (48%) (including 7/8 teenagers [87%]) reached Tanner Stage 5 by EoS.

**Fig. 2:**
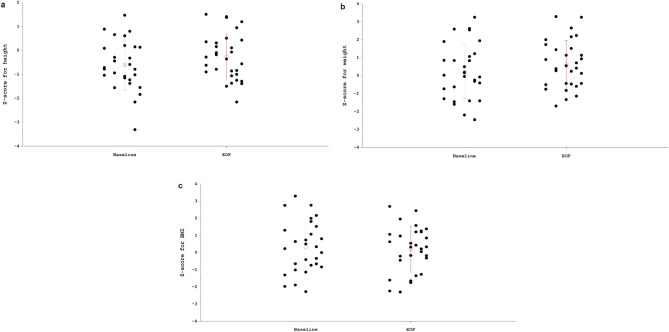
Comparison of mean height Z-score (**a**), weight Z-score (**b**), and BMI Z-score (**c**) data at baseline (M1) and EoF (post-hoc analysis, paired t-test). BMI, body mass index; EoF, End of Follow-up; M, Month

Mean height Z-score significantly increased between baseline and EoF (-0.6 ± 1.0 to -0.3 ± 1.0, *p* = 0.03) (Fig. 2A) (descriptive analysis by age group and in all patients in Supplemental Figure S1), whilst mean weight and BMI Z-scores remained stable (p = NS) (Fig. 2B and C) (Table 1).

**Fig. 3:**
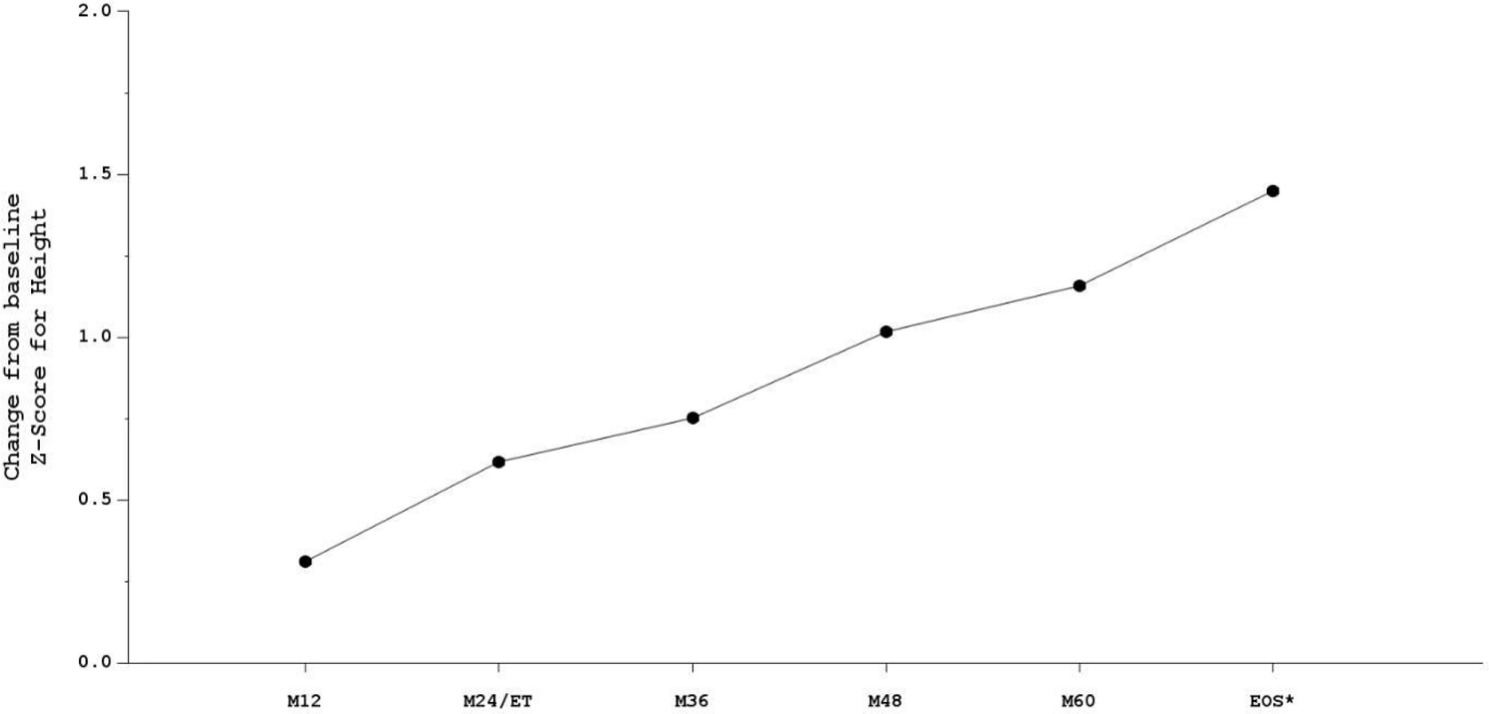
Evolution of mean height Z-score in patients with abnormal EAS at baseline (post-hoc analysis, repeated measure model on change adjusted on baseline) (*n* = 6), *p* < 0.001). EAS, estimated adult stature; EoS, End of Study; ET, early termination visit; M, month; n, number of evaluated patients

Overall, most patients had a normal EAS at baseline. The proportion of patients with a normal EAS remained stable throughout the study from 20/26 patients (77%) at baseline to 21/26 patients (81%) at EoF, (p = NS) (Table 1). Among the 6 patients with abnormal EAS at baseline, 4 patients reached a normal EAS at EoF and, in these 6 patients, the change adjusted on baseline of Z-score for height significantly improved over the study period (*p* < 0.001) (Fig. 3).

### Evolution of kidney function

**Fig. 4:**
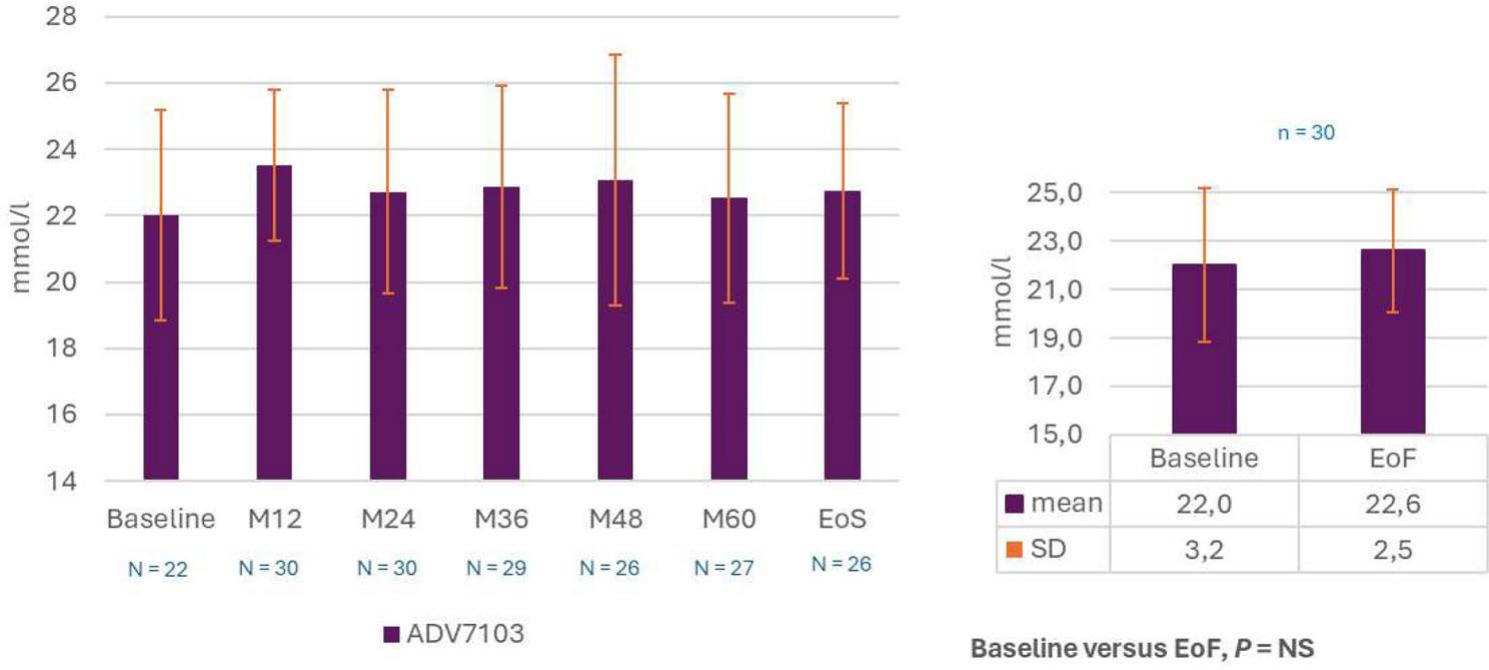
Mean plasma bicarbonate levels over time (descriptive analysis) and comparison EoF versus Baseline (post-hoc analysis, paired t-test). EoF, End of Follow-up; EoS, End of Study; M, month; N/n, number of evaluated patients; NS, non-significant; SD, standard deviation

**Fig. 5:**
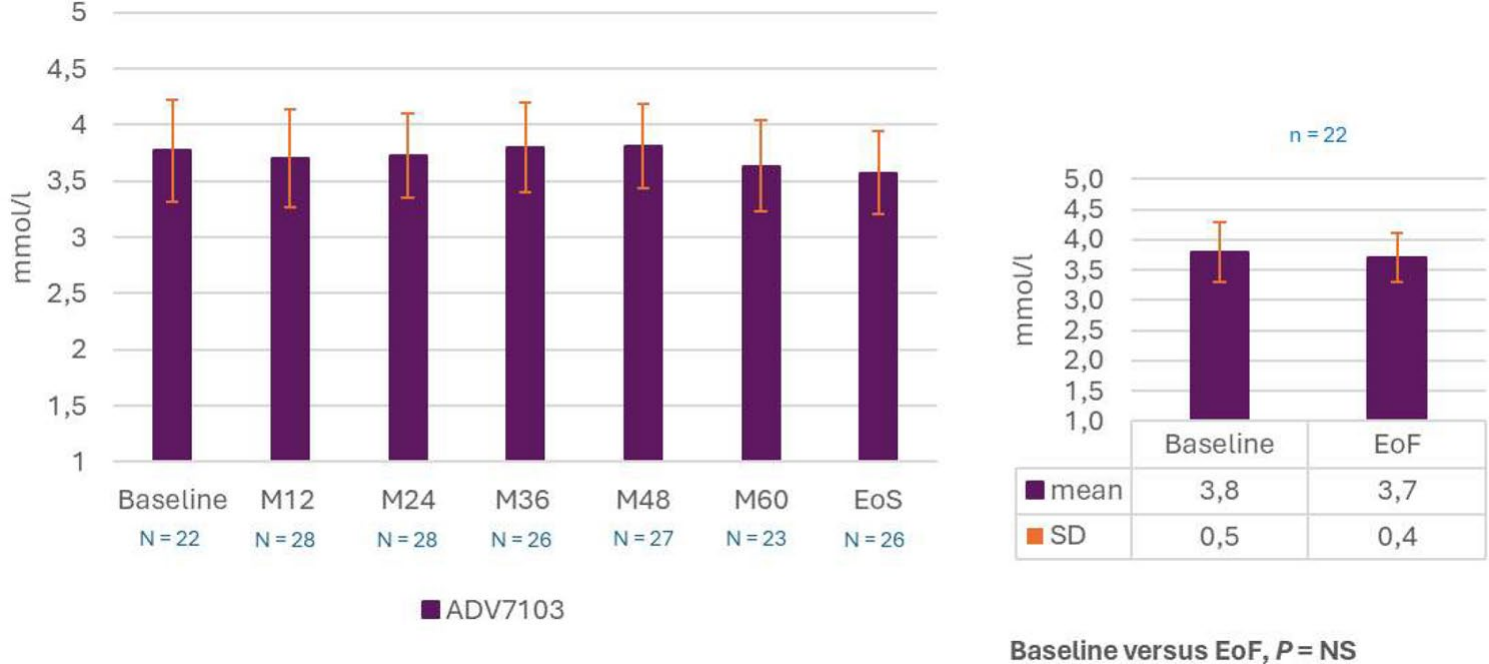
Mean plasma potassium levels over time (descriptive analysis) and comparison EoF versus Baseline (post-hoc analysis, paired t-test). EoF, End of Follow-up; EoS, End of Study; M, month; N/n, number of patients; NS, non-significant; SD, standard deviation

Mean plasma bicarbonate levels remained stable throughout the 6-year follow-up from 22.0 ± 3.2 at baseline to 22.6 ± 2.5 mmol/L at EoF, p = NS (Fig. 4). In the subset of patients with blood sampling performed before study treatment intake, the same evolution profile was shown. Mean plasma potassium levels were also stable over the study duration, from 3.8 ± 0.5 at baseline to 3.7 ± 0.0.4 mmol/L at EoF, p = NS (Fig. 5).

Mean eGFR values remained stable over time, from 105 ± 17 at baseline to 104 ± 20 mL/min/1.73 m [2] at EoF, p = NS (Table 1).

Among 23 patients with an eGFR measurement, 20 patients had CKD stage 1 at baseline (87%) and 17 patients (74%) at EoF. In addition, 3 patients had CKD stage 2 at baseline (13%) and 6 patients at EoF (26%). There was no statistical difference in CKD stages breakdown between baseline and EoF (p = NS) and no patient reached CKD stage 3 to 5 during the study (Table 1).

Urinary ratios associated with the risk of nephrolithiasis/nephrocalcinosis (UCa/UCr, UCi/UCr and UCa/UCi) were not significantly different between baseline and EoF (p = NS) (Table 1).

The percentage of patients with urine crystals tended to decrease (53% at baseline and 35% at EoF, p = NS). There was no significant increase in the occurrence of ACCP crystals, 47% patients at baseline versus 35% patients at EoF, p = NS (Table 1).

The proportion of patients with urinary pH in the 7–8 range was stable across the study (70% at baseline and EoF). The proportion of patients with urine pH < 7 or > 8 respectively varied from 9% to 22% at baseline to 0% and 30% at EoF, p = NS. So, there was no impact on ACCP crystals presence despite some patients with urinary pH > 8 (Table 1).

### Kidney ultrasounds / CT scans

From the descriptive analysis, there were 6/29 (21%) patients and 11/25 (44%) patients with nephrolithiasis, respectively at baseline and EoS. Only one case of surgical procedure for kidney stone removal during the study course was reported.

From the descriptive analysis, nephrocalcinosis was present in 25/29 patients (86%) at baseline and in 23/25 patients (92%) at EoS.

### Evolution of bone biomarkers

None of the adults were diagnosed with osteomalacia throughout the study. Rickets was reported in 1 infant at baseline and 4 pediatric patients at Month 36 (2 children and 2 teenagers). From Month 60, rickets were no longer reported in patients.

PTH levels remained in the normal range over 48 months: 15.6 ± 8.2 to 16.6 ± 6.6 ng/L at baseline and Month 48, respectively (Table 1).

Concerning bALP levels, most patients (from 75 to 96%, according to the study visit) had levels in the normal range over 48 months and mean bALP Z-score was maintained from baseline to Month 48 in the overall population, p = NS. Mean serum phosphate Z-score significantly decreased over 48 months from − 0.7 ± 0.7 to -1.4 ± 1.1, *p* = 0.005 (Supplemental Figure S2). Mean TmP/GFR also significantly decreased from 1.5 ± 0.2 at baseline to 1.3 ± 0.3 mmol/L at EoF, *p* = 0.001. Mean circulating calcium levels slightly increased from 2.38 ± 0.10 at baseline to 2.42 ± 0.08 mmol/L at EoF, *p* = 0.048. Mean serum 25-OH-vit-D levels were low during the study (55.0 ± 33.7 at baseline and 51.0 ± 19.2 nmol/L at Month 48) while mean serum 1-25-OH-vit-D levels were in the normal ranges (164.6 ± 54.1 at baseline and 158.6 ± 36.3 pmol/L at Month 48), p = NS (Table 1).

### Evolution of bone mineral density

**Fig. 6:**
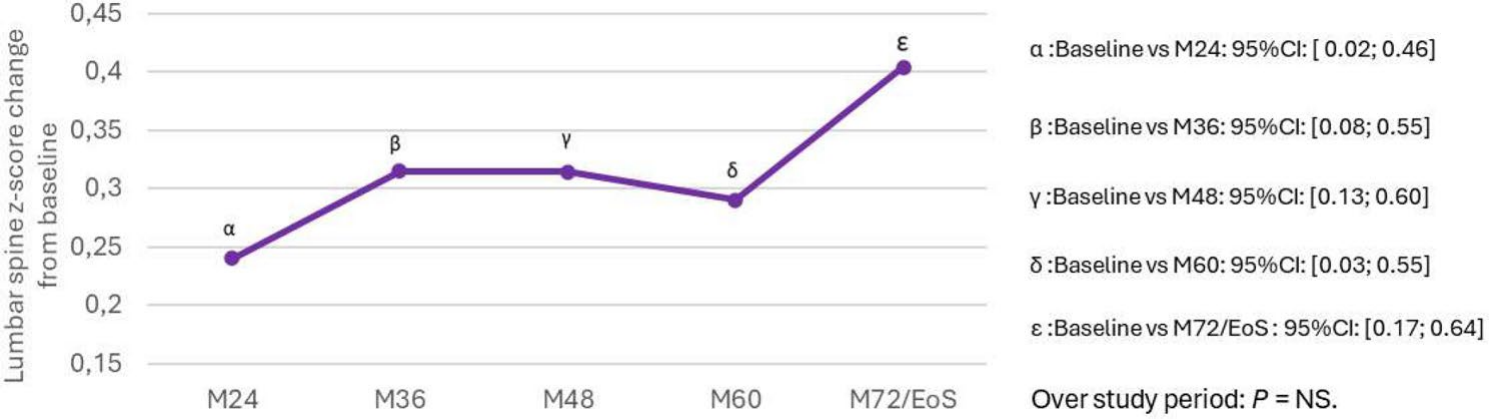
Evolution of mean lumbar spine BMD Z-score: mean change from baseline in all patients (post-hoc analysis, repeated measure model adjusted on baseline). BMD, bone mineral density; CI, confidence interval; ET, early termination visit; M, month; NS, non-significant; SD, standard deviation

A significant improvement in mean lumbar spine BMD Z-score was observed from baseline (-1.1 ± 1.0) to EoF (-0.8 ± 1.0), *p* = 0.005. Lumbar spine BMD Z-scores were normal in most patients from baseline (71%) to EoF (87%) (Table 1). Over the study duration, lumbar spine BMD Z-score evolution was not statistically significant, while the change from baseline to each yearly visit was statistically significant using the same statistical test (Fig. 6).

**Fig. 7:**
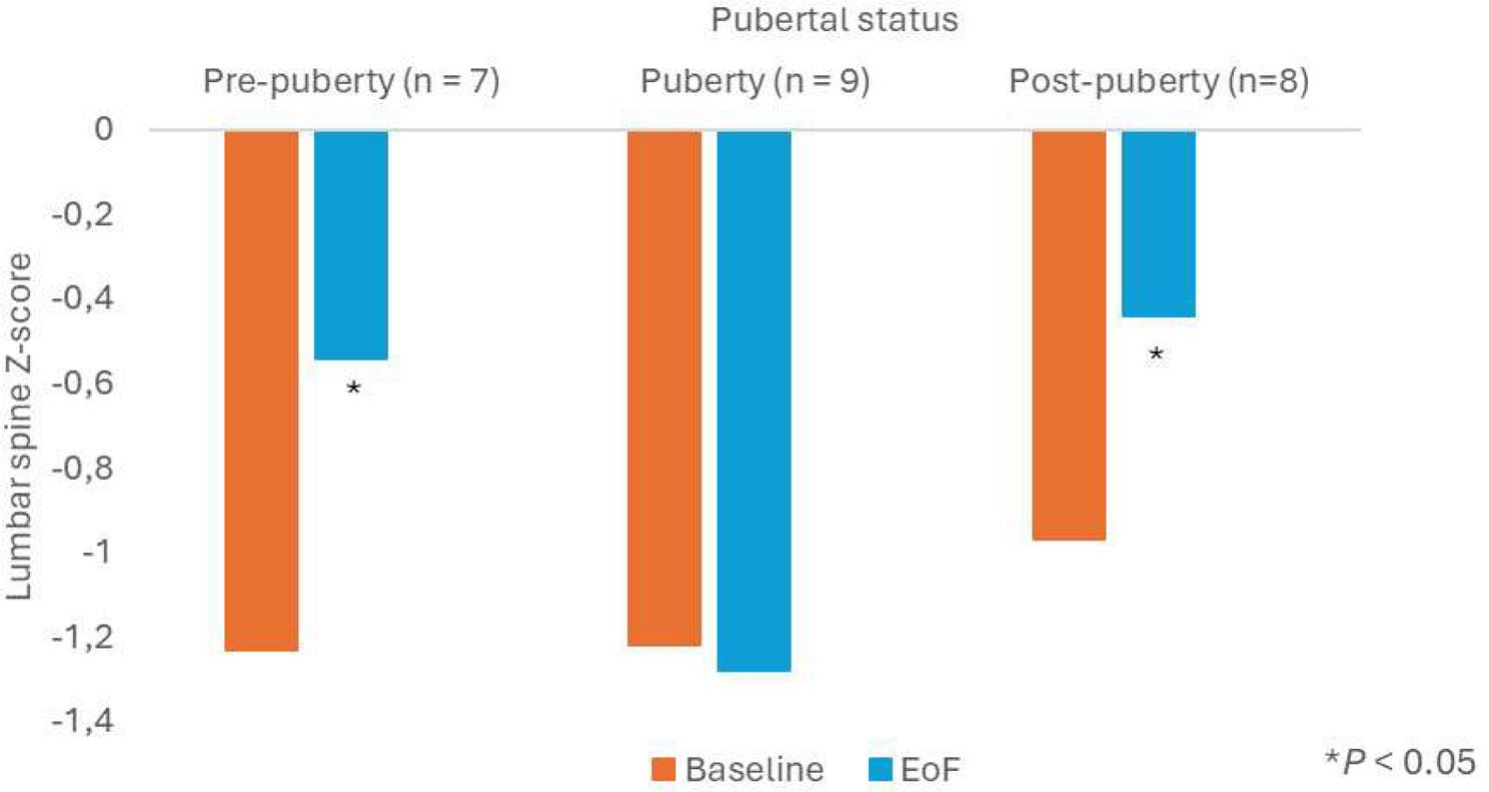
Mean lumbar spine BMD Z-score at baseline and EoF by pubertal status according to baseline age (post-hoc analysis, paired t-test). BMD, bone mineral density; EoF, End of Follow-up; n, number of patients

Mean lumbar spine BMD Z-score varied according to patients’ pubertal status with a significant improvement from baseline to EoF in pre-and post-pubertal patients while it was maintained in patients in pubertal phase: -1.2 ± 1.3 at baseline and − 0.5 ± 0.9 at EoF, *p* = 0.035 in pre-pubertal patients, -1.0 ± 1.0 at baseline and − 0.4 ± 0.8 at EoF, *p* < 0.001 in post-pubertal patients and − 1.2 ± 0.9 at baseline and − 1.3 ± 1.0 at EoF, p = NS in pubertal patients (Fig. 7).

### Sibnayal^®^ dose and compliance

The overall mean dose prescribed evolved from 3.4 ± 1.7 at baseline to 2.3 ± 1.1 mEq/kg/d at EoS (Supplemental Figure S3).

The compliance rate was good or excellent in 58–83% patients, depending on the visit considered. At EoS, compliance rate was good to excellent for 16/27 patients (60%). Compliance was lower in teenagers whose compliance rate was poor at EoS for 4/7 teenagers (57%) compared to 0/5 adults, 1/12 children (8%) and 0/3 infants (Supplemental Figure S4).

## Discussion

In dRTA patients, metabolic acidosis control is the main goal to prevent short and long-term complications of the disease [28–31]. This objective can be highly challenging to attain for patients carrying H^+^-ATPase variants [32, 33]as they are responsible for the earliest manifestations of the disease. Thus, the current challenge for pediatric and adult nephrologists in dRTA is to improve the proportion of patients displaying adequate metabolic control and to have robust outcome data to prevent long-term complications, especially on growth, bone metabolism and kidney function.

Few long-term outcome data have been reported to date. A collaborative work between ERKNet and the European Renal Association collecting data from a large European multinational cohort of dRTA patients is ongoing [34]. Lopez-Garcia et al. reported data from a large cohort of 340 patients (29 countries) with a median age at last follow-up of 11.0 (0.0–70.0) years [5]. The median prescribed dose of alkali treatment was 1.9 mEq/kg/d. Only 51% of patients achieved adequate metabolic control under alkali treatment, keeping in mind that higher bicarbonate plasma levels were associated with better growth and kidney function. This work was performed in the context of real-world setting where patients could be less motivated to take their medication conversely to patients included in a clinical study.

Sibnayal^®^ is a prolonged-release formulation of potassium citrate and potassium bicarbonate with a twice daily administration, approved by the European Medical Agency in April 2021 in the treatment of patients with inherited or acquired dRTA, aged one year and older. Sibnayal^®^ has been developed over the last decade from a collaboration between French physicians and a pharmaceutical company in response to a direct request from clinicians. The pivotal B21CS study showed that plasma bicarbonate levels and response rate were significantly higher with Sibnayal^®^ than with SoC [7]. In addition, over 24 months, a sustained control of metabolic acidosis was shown together with a good treatment acceptability and an improvement of Quality of Life (QoL) (quantitative assessment) [8]. In addition, a long-term QoL analysis (qualitative assessment) was done using semi-structured interviews with patients and/or parents after at least 48 months of treatment, with an average of 5 years. Results were previously published and showed a high level of patients/parents’ satisfaction [35].

The good safety profile over 24 months previously published was confirmed over 6 years and no additional safety or tolerability concerns associated to the use of Sibnayal^®^ were raised. No pattern of increasing adverse events (treatment-related or not) was observed with increasing duration of exposure to the treatment.

The present study also confirms the long-term efficacy of Sibnayal^®^ to maintain normal plasma bicarbonate levels. A total of 73% of patients displayed normal plasma bicarbonate after an average 6-year follow-up. As shown in Lopez-Garcia’s cohort [5] adequate metabolic control is significantly linked with growth improvement. With Sibnayal^®^, mean height Z-score significantly increased without BMI increase. We didn’t perform any sub-analysis excluding adult patients. Nevertheless, it should be noticed that the improvement of height in the adult group may be explained by the mean age of this group at inclusion (19.0 ± 0.1 years). Their height improvement was only observed during the first 24 months of the study. We hypothesize these Tanner 5 patients had a growth retardation at inclusion which was not captured as bone age was not measured in our study.

In our study, one patient had a very low height Z-score at baseline (-3.3 SD) which could have influenced the statistical significance of the results. Nevertheless, a significant growth improvement was observed in patients with abnormal EAS at baseline while patients with normal EAS at baseline had a stable height over the study. Our data confirmed that adequate metabolic acidosis control allowed a better growth prognosis for patients including young adult patients.

Kidney function in patients with dRTA declines significantly earlier than in general population, CKD affecting indeed at least a third of dRTA patients from the second decade of life [3–5, 32].

When considering the comparable age patient subset from Lopez-Garcia’s cohort [5] (*n* = 167, mean age: 15.0 ± 5.0 years): 45.5% of their patients were CKD stage ≥ 2 versus 26.0% in our study. For the first time, we report a promising stabilization of kidney function during the long-term observation period in dRTA patients receiving Sibnayal^®^.

In our study, and as already reported in retrospective studies [3–5, 32, 36] nephrocalcinosis was reported in most patients at baseline, leading to difficult interpretation of Sibnayal^®^ effect on this parameter. Over the study duration, calciuria and citraturia were maintained in normal range, in 90% and 45% of patients, respectively (stable risk of lithogenesis). So, we could expect a decrease in intra-tubular crystallization and of calcium deposits in the kidney which could protect renal function [37, 38].

As reported by Lopez-Garcia, nephrolithiasis was diagnosed in 21% of our patients at baseline and tended to increase with time, as 11/25 (44%) patients, had a diagnosis of nephrocalcinosis at EoS. There was only one case of kidney stone removal during the 6-year study, while this event has been reported as the most frequent complication during the dRTA patient management [39].

An additional major concern for physicians is the bone and mineral management of dRTA patients as there is few published data about the correction of bone parameters with alkali treatments. In a small cohort of adult patients with secondary dRTA (*N* = 14), Domrongkitchaiporn et al. reported osteopenia, fractures and significantly low BMD [40, 41]. After one year of alkali therapy, they showed a significant increase in BMD and a normalization of bone formation rate. They hypothesized that chronic metabolic acidosis results in a suppression of bone formation and resorption, as previously shown in experimental papers demonstrating a deleterious effect of acidosis both on osteoblastic differentiation and osteoclastic differentiation/activity [42–44, 32]. In our study, 1- 25-OH-vit-D, PTH and most bone markers remained in the normal range, specifically bALPs, while a significant decrease in mean serum phosphate Z-score was observed. Without having measured FGF 23 serum level, it is difficult to suggest a pathophysiological mechanism. Nevertheless, the significant increase in bone mass is consistent with an improvement of bone modeling rate and mineralization. This is supported by a continuous and significant improvement of lumbar spine BMD Z-score, expressed as change from baseline. It can nevertheless also be argued that serum phosphate levels decrease with age, and particularly during childhood and adolescence, but the data were analyzed using standardization for age to avoid this age-specific effect, so that the decreased phosphate Z-score during the study remains intriguing. In contrast, the decrease in absolute TmP/GFR is expected in view of the kinetics of TmP/GFR during puberty and early adulthood.

These positive long-term effects of Sibnayal^®^ are linked to its efficacy but also likely to the good compliance to treatment maintained throughout the follow-up except in teenagers where high prevalence of poor adherence to chronic treatments is well-known [45]. This age group is known to require specific multidisciplinary teams, including social workers, psychologists, specific transition programs [46].

An experience with Sibnayal^®^ in the hospital real-world setting was recently published [47]. In 13 pediatric patients with dRTA, it shows an awaited efficacy of Sibnayal^®^ (*N* = 10) with an adequate metabolic control observed in 89% of patients versus 78% in patients receiving SoC (*N* = 3) (p = NS). Concerning SoC treatment, these results are better than those observed in the European cohort [5]. This may reflect that treatment occurred in a dedicated clinic for tubular diseases in a large tertiary centre, which is not fully representative for dRTA patients [47].

The main limitation of our clinical study was the absence of a direct comparator during follow-up. However, a long-term randomized controlled trial was not considered feasible (nor ethical) in a rare disease such as dRTA. The set-up of the European dRTA registry will further consolidate the long-term evolution of dRTA patients based on alkali treatments [34]. Our study included a small number of patients as dRTA is a very rare disease. As a consequence, we present results in the whole population. In addition, the long-term follow-up of the patients in our study along with the use of paired t-test for most of statistical analyses of exploratory efficacy endpoints resulted in a low number of patients for some of them. This was mostly the case for bone biomarkers analyses.

Another limitation is the absence of morpho-constitutional analysis of stones and not enough CT imaging performed to detect and follow nephrocalcinosis and nephrolithiasis.

In the long-term, treatment with Sibnayal^®^ allows the control of metabolic acidosis and the prevention of dRTA complications with a good safety and tolerability profile. Sibnayal^®^ significantly improves growth and lumbar spine BMD in pediatric and young adult dRTA patients. Most importantly, kidney function is preserved over 6 years on average. Overall, these results contribute to improving the management and outcomes of this orphan disease.

## Electronic supplementary material

Below is the link to the electronic supplementary material.

Supplementary Material 1

## Data Availability

The data that support the findings of this study are available from ADVICENNE, but restrictions apply to the availability of these data, which were used under license for the current study, and so are not publicly available. Data are however available from the authors upon reasonable request and with the permission of ADVICENNE.
