# Supplementary material for: 6-year treatment follow-up with an extended-release alkaline formulation (Sibnayal®) in primary distal renal tubular acidosis
Source: Orphanet J Rare Dis. 2025 Aug 13;20:431. doi: 10.1186/s13023-025-03953-4 (PMC12351860; doi:10.1186/s13023-025-03953-4)

Supplementary material

**Table S1.** Treatment-emergent AEs (related TEAEs) related to treatment reported during the 6-year treatment with Sibnayal®.

| Body system  Preferred term | N related TEAEs | Patients with at least one related-TEAE, all patients (N = 30) | |
| --- | --- | --- | --- |
|  |  | n | % |
| All | 13 | 6 | 20.0 |
| Gastrointestinal disorders | 11 | 5 | 16.7 |
| Abdominal pain | 2 | 2 | 6.7 |
| Abdominal pain upper | 2 | 2 | 6.7 |
| Diarrhea | 3 | 1 | 3.3 |
| Dyspepsia | 2 | 2 | 6.7 |
| Gastrointestinal disorder | 1 | 1 | 3.3 |
| Gastrointestinal pain | 1 | 1 | 3.3 |
| Metabolism and nutrition disorders | 1 | 1 | 3.3 |
| Hypokalemia | 1 | 1 | 3.3 |
| Kidney and urinary disorders | 1 | 1 | 3.3 |
| Nephrolithiasis | 1 | 1 | 3.3 |

N, number of evaluated patients; n, number of patients; TEAE, treatment-emergent adverse event.

**Supplementary Figure S1.** Evolution of mean height Z-score by age group and in all patients (descriptive analysis).


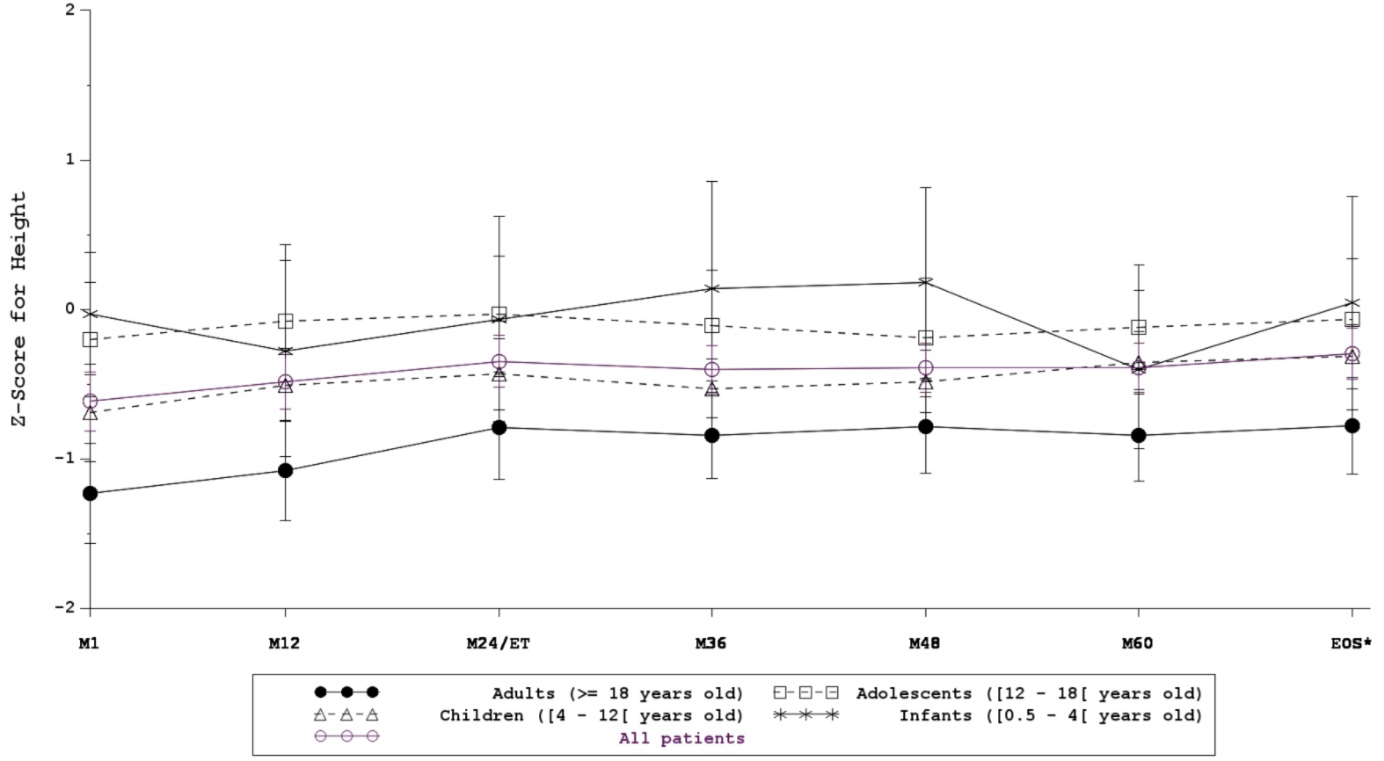


**Supplementary Figure S2.** Evolution of mean plasma phosphate Z-score by age group and in all patients (descriptive analysis).


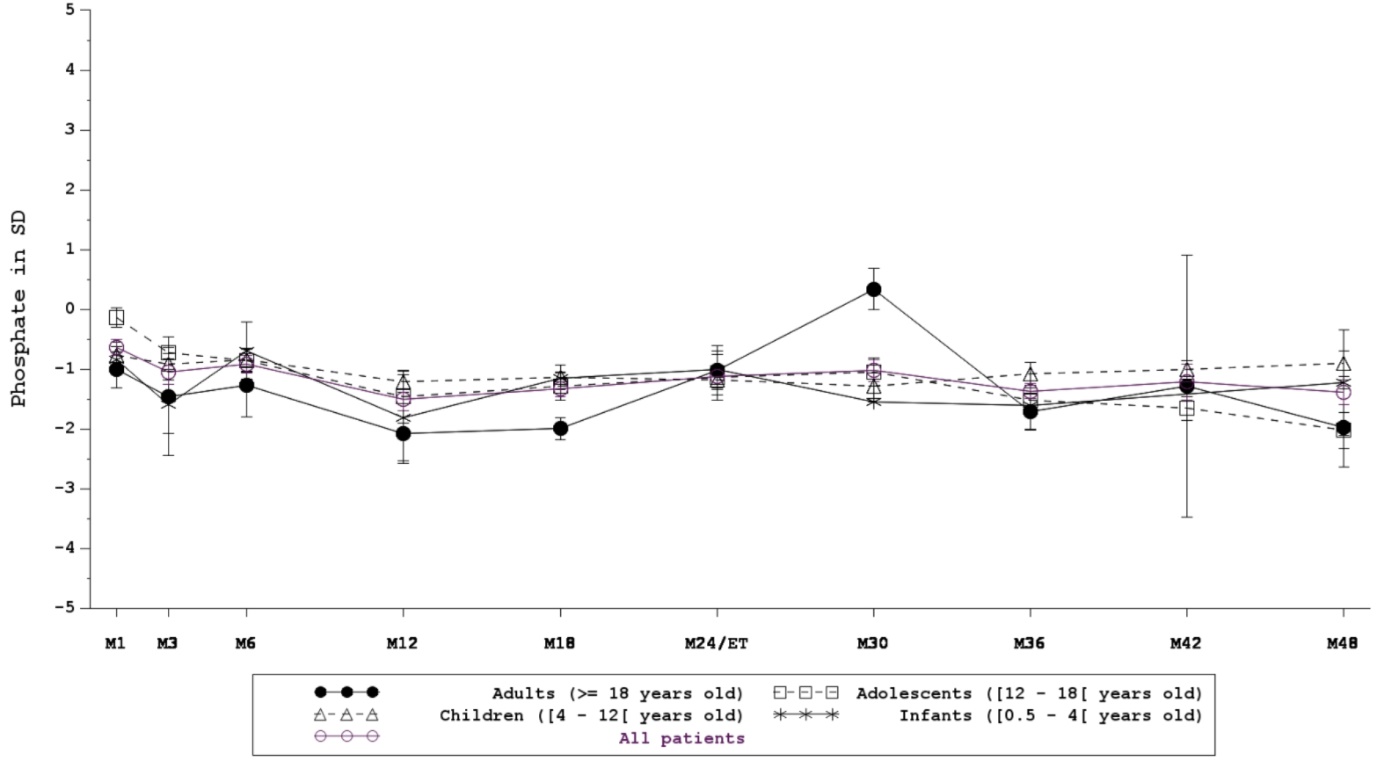


**Supplementary Figure S3.** Mean prescribed Sibnayal® dose over time (mEq/kg/d) by age group and in all patients (descriptive analysis). Only the dose administered at the beginning of the interval was used for the analysis.


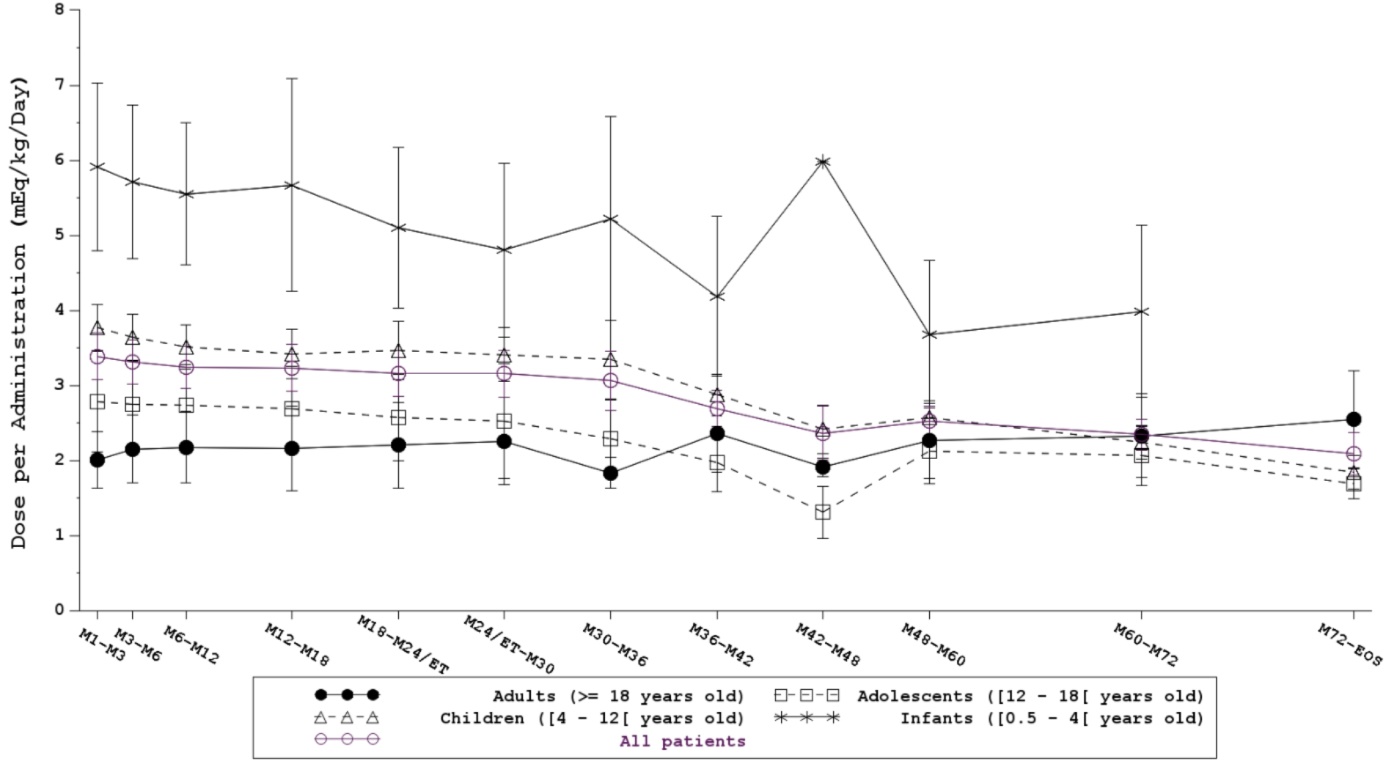


**Supplementary Figure S4.** Long-term compliance in all patients (descriptive analysis).


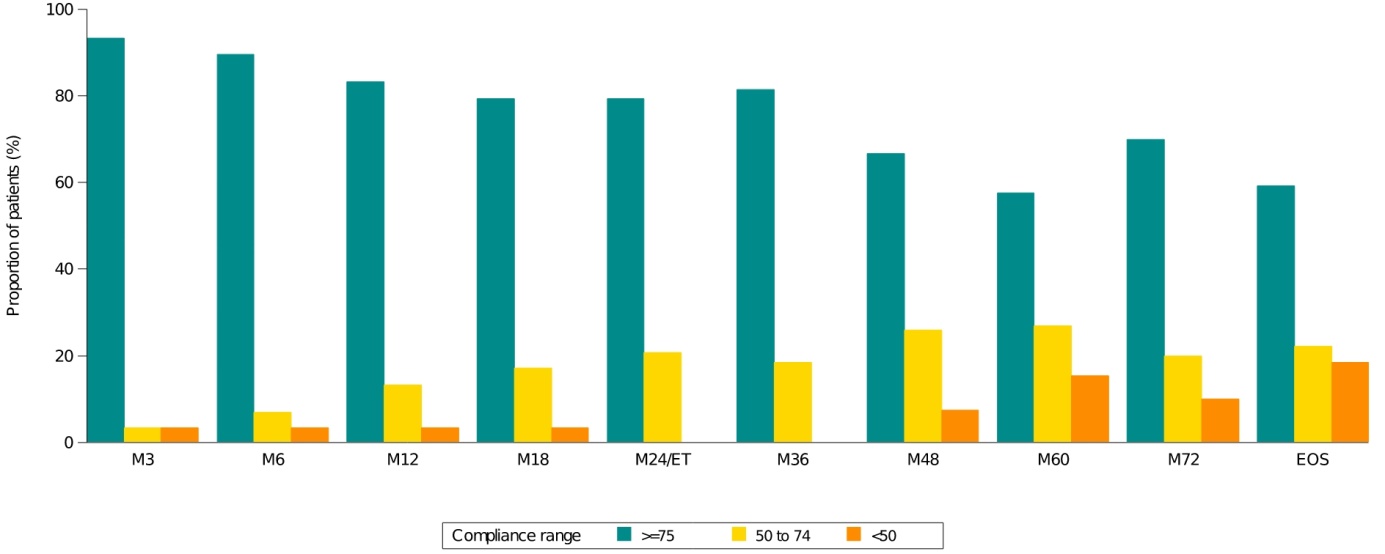

Supplement: Supplementary file 1 — Supplementary Material 1 [file 13023_2025_3953_MOESM1_ESM.docx]
